# A virus-free cellular model recapitulates several features of severe COVID-19

**DOI:** 10.1038/s41598-021-96875-7

**Published:** 2021-09-01

**Authors:** Giovanni Lavorgna, Giulio Cavalli, Lorenzo Dagna, Silvia Gregori, Alessandro Larcher, Giovanni Landoni, Fabio Ciceri, Francesco Montorsi, Andrea Salonia

**Affiliations:** 1 Division of Experimental Oncology/Unit of Urology, URI, IRCCS Ospedale San Raffaele, Milan, Italy; 2 University Vita-Salute San Raffaele, Milan, Italy; 3 Unit of Immunology, Rheumatology, Allergy and Rare Diseases (UnIRAR), IRCCS San Raffaele Scientific Institute, Milan, Italy; 4 San Raffaele Telethon Institute for Gene Therapy (SR-TIGET), IRCCS Ospedale San Raffaele, Milan, Italy; 5 Anesthesia and Intensive Care Department, IRCCS Ospedale San Raffaele, Milan, Italy; 6 Hematology and Bone Marrow Transplant Unit, IRCCS Ospedale San Raffaele, Milan, Italy

**Keywords:** Systems biology, Evolvability, Diseases, Infectious diseases, Viral infection

## Abstract

As for all newly-emergent pathogens, SARS-CoV-2 presents with a relative paucity of clinical information and experimental models, a situation hampering both the development of new effective treatments and the prediction of future outbreaks. Here, we find that a simple virus-free model, based on publicly available transcriptional data from human cell lines, is surprisingly able to recapitulate several features of the clinically relevant infections. By segregating cell lines (n = 1305) from the CCLE project on the base of their sole angiotensin-converting enzyme 2 (ACE2) mRNA content, we found that overexpressing cells present with molecular features resembling those of at-risk patients, including senescence, impairment of antibody production, epigenetic regulation, DNA repair and apoptosis, neutralization of the interferon response, proneness to an overemphasized innate immune activity, hyperinflammation by IL-1, diabetes, hypercoagulation and hypogonadism. Likewise, several pathways were found to display a differential expression between sexes, with males being in the least advantageous position, thus suggesting that the model could reproduce even the sex-related disparities observed in the clinical outcome of patients with COVID-19. Overall, besides validating a new disease model, our data suggest that, in patients with severe COVID-19, a baseline ground could be already present and, as a consequence, the viral infection might simply exacerbate a variety of latent (or inherent) pre-existing conditions, representing therefore a tipping point at which they become clinically significant.

## Introduction

A highly variable spectrum of clinical manifestations accompanies the new severe acute respiratory syndrome coronavirus 2 (SARS-CoV-2)-induced disease (COVID-19), ranging from mild respiratory illness to severe pneumonia, multiorgan failure and death. Apparently, SARS-CoV-2 is strongly related to SARS-CoV, which caused the well-known severe acute respiratory syndrome almost two decades ago^1^. From a mechanistic point of view, there is overwhelming evidence indicating that SARS-CoV-2 enters cells by binding to the angiotensin-converting enzyme 2 (ACE2)^2^. Of importance, ACE2 activity is both necessary and sufficient for viral infection. Indeed, a monoclonal antibody directed against ACE2 blocks viral infection in permissive cells^3^, whereas exogenous expression of human ACE2 allows SARS-CoV infection in non-human cells^4^. Additionally, it has been shown that human HeLa cells overexpressing ACE2 from a variety of species become amenable to SARS-CoV-2 infection and replication^5^. Moreover, ACE2 levels can also influence the degree of disease progression: in a mice cohort engineered to express different levels of human ACE2, animals expressing the highest levels of ACE2 mRNA displayed the worst survival upon viral infection^6^. Therefore, it is likely that the amount of ACE2 expression has a significant role on susceptibility to SARS-CoV-2. Along this line, a transcriptional analysis in the lung adenocarcinoma dataset of The Cancer Genome Atlas (TCGA) revealed that ACE2 expression, while not affected by the tumor status, was positively correlated with age^7^; this latter finding combines well with the observation that elderly people are more vulnerable to SARS-CoV-2^8–10^.

As a whole, ACE2 appears to be a key player in mediating the severity of SARS-CoV-2 infection. On this premise, we built a ‘guilt-by-association’ model^11^ by determining differential pathway expression in low- and high-expressing ACE2 cell lines from the Cancer Cell Line Encyclopedia (CCLE) project. As a result, we found that, even in the absence of a viral infection, ACE2 overexpressing cell lines displayed several cell-intrinsic characteristics predisposing to the development of a more severe disease phenotype upon infection. Of note, we also found a strong sex-related bias disfavoring males in terms of strength of the activated or repressed pathways. Likewise, several compounds appeared to be potentially repurposable for disease treatment, with a few of them already in use or considered for the treatment of patients with COVID-19.

## Results

### Study design and model building: the link to ACE2 overexpression

**Figure 1:**
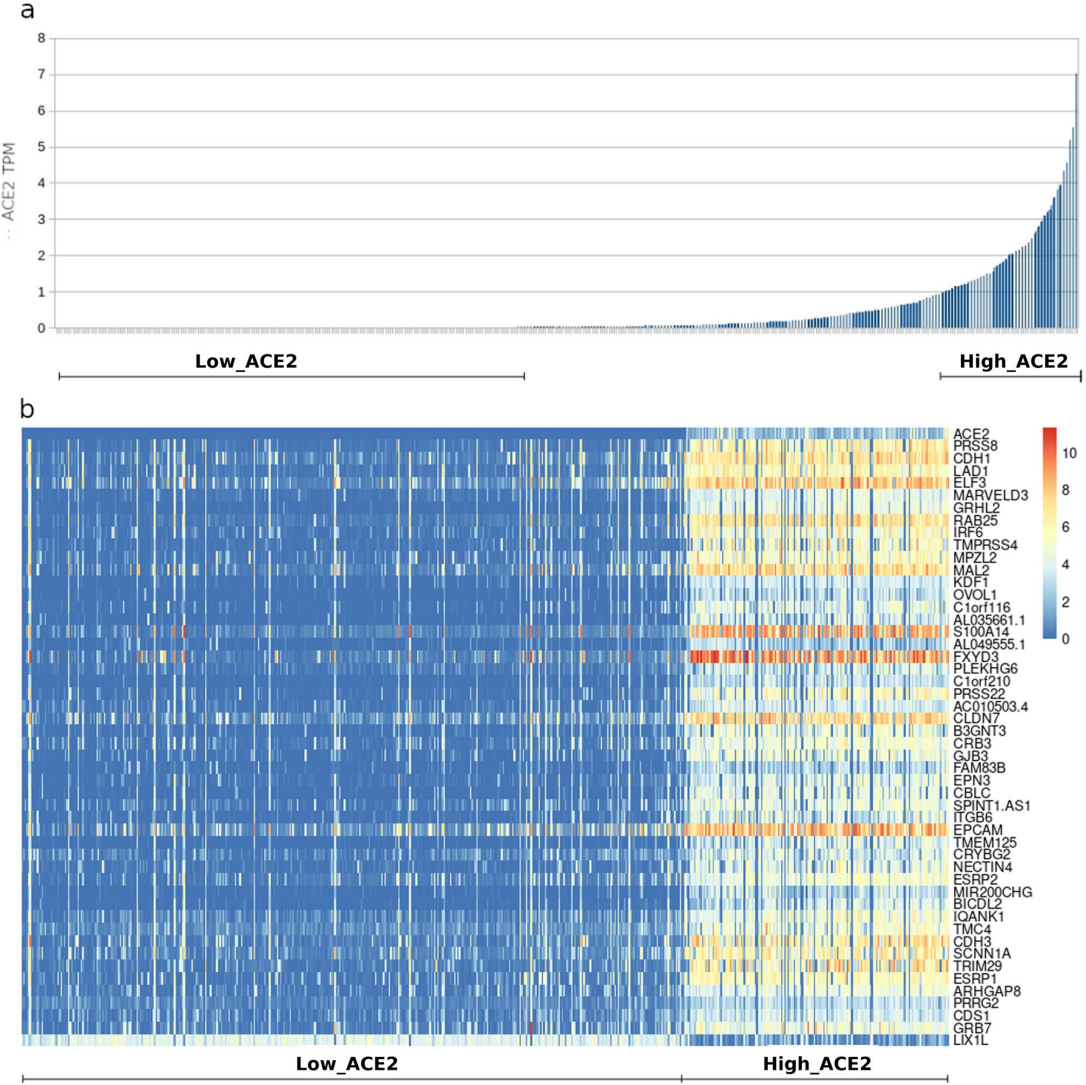
Building a virus-free COVID-19 disease model based on differential ACE2 expression in human cell lines. (**a**) 1305 cell lines from the Cancer Cell Line Encyclopedia (CCLE) project were sorted on the base of their ACE2 TPM (Transcripts Per Million) content. Cell lines displaying a ACE2 TPM value equal to 0 (Low ACE2) or greater than 1 (High ACE2) were grouped. (**b**) Top 50 differentially expressed transcripts between Low ACE2 vs. High ACE2 cell lines.

RNA-seq data from 1305 cell lines of the CCLE project were sorted on the basis of their ACE2 content. Then, differentially expressed transcripts between cell lines displaying an ACE2 TPM (Transcripts Per Million) value greater than 1 (‘High_ACE2’) or equal to 0 (‘Low_ACE2’) were calculated (Fig. 1a). Out of 58,676 transcripts, differential expression analysis identified 602 upregulated and 1488 downregulated transcripts in 'High_ACE2' samples (see Supplementary Table 1). A heatmap of the top 50 differentially expressed transcripts is shown in Fig. 1b. Among the upregulated genes, we found genes already known to be involved in the COVID-19 pathogenic mechanisms, like TMPRSS4, that has been shown to promote infection of SARS-CoV-2 in human small intestinal enterocytes along with TMPRSS2 gene^12^. Similarly, a Gene Ontology analysis (Supplementary Table 2), found that 16 genes (SCNN1A, MAL2, ACE2, CRB3, EPN3, IRF6, CDH1, TMC4, NECTIN4, RAB25, C1orf116, S100A14, FXYD3, EPCAM, LAD1, PRSS8) were related to cellular exosomes, vesicles that may enhance the viral spread, as well as they were able to transfer receptors, such as ACE2, making recipient cells susceptible to virus docking^13^. In the same plot, 15 genes (GRHL2, ITGB6, CLDN7, NECTIN4, CRB3, PLEKHG6, TRIM29, CDH3, GRB7, EPCAM, KDF1, GJB3, IRF6, CDH1, MARVELD3) were instead found to be involved in cell-junctions, a cellular component widely used by viruses to transit through the epithelia^14^. Among the individual top-50 differentially expressed genes, there were several other genes that, while not having yet been reported to have a clear clinical association with COVID-19, possessed a function suggesting a potential role in the disease. For instance, CLDN7 is an epithelial barrier expressed gene^15^ already known to be related to ACE2 expression in single-cell sequencing experiments^16^. As ACE2 expression is increased by smoking in the respiratory tract, CLDN7 could be implied in the complex interaction between SARS-CoV-2 infection and smoking^17^. Along the same line, the upregulation of the S100A14 gene was also suggestive of a possible involvement in the disease, since this gene encodes for a DAMP (Damage-Associated Molecular Patterns), molecules released by damaged cells. Although DAMPs help the host defence, they might contribute to a pathological immune response^18^, strikingly paralleling the observation that COVID-19 morbidity and mortality has been associated since the very beginning to a drastic systemic inflammatory response^1,19^. It should be noticed that the involvement of DAMPs in the disease could be even bigger, as several other members of the S100 family, S100P, S100A9, S100A8, S100A7, S100A16, S100A2, S100A11, S100A10 and S100A6, are upregulated in ACE2 overexpressing cells as well, as shown in Supplementary Table 1. Taken together, these data suggest that ACE2 overexpressing cells might possess additional features that make them even more amenable to SARS-CoV-2 infection, propagation and damage.

In order to further characterize pathways involved with high ACE2 expression levels, we performed a variety of Gene Set Enrichment Analysis (GSEA)^20^ using the Kegg, the Reactome and the Hallmarks datasets from the Molecular Signature Database (MSigDB)^21,22^, along with the Gene Ontology Biological Processes database and the Drug Signature Database (DSigDB)^23^. As a result, 178 gene-sets were found to be differentially expressed (Supplementary Table 3).

### A hyperinflammatory/immune response is associated to high ACE2 expression

**Figure 2:**
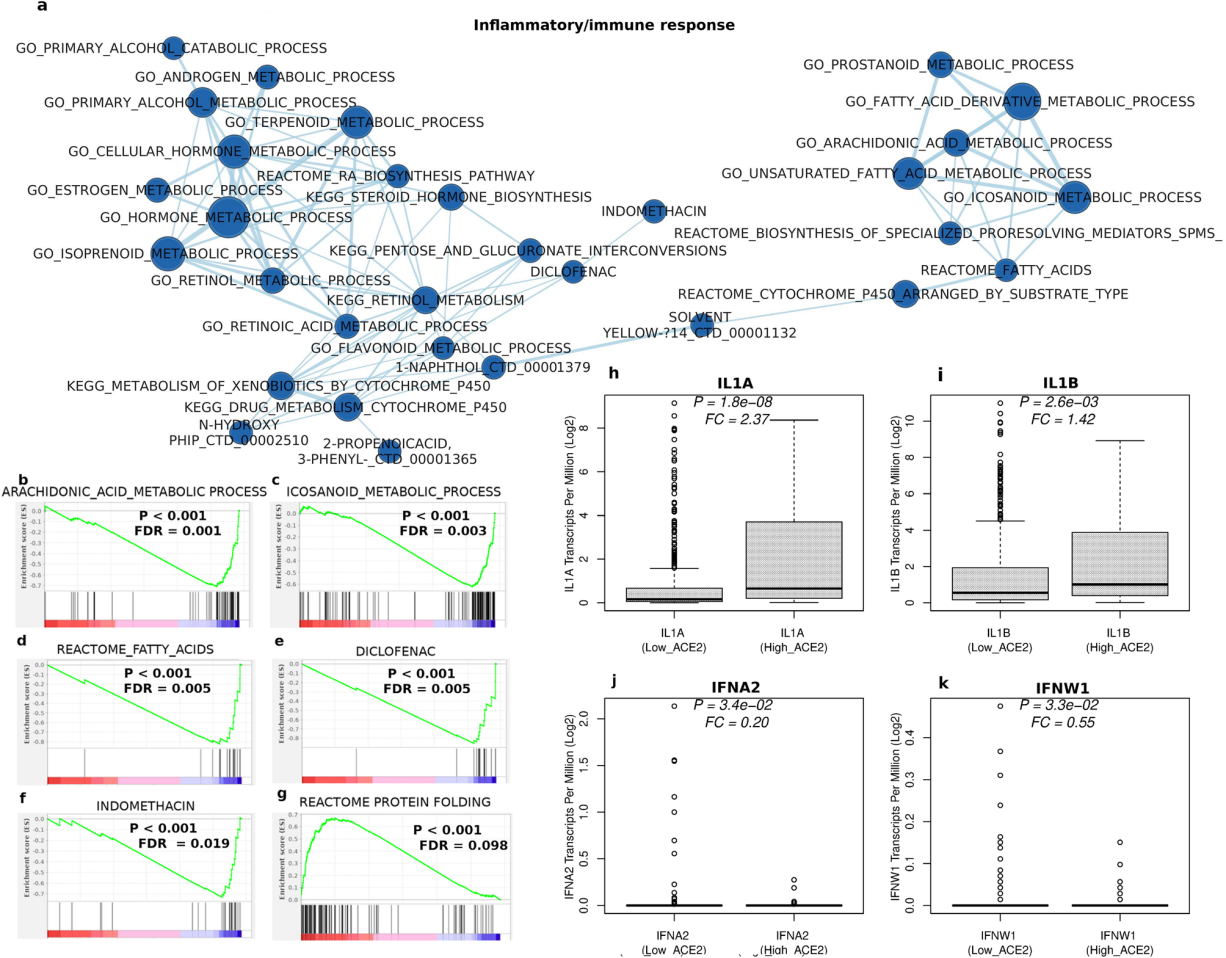
ACE2 overexpressing cell lines mimic host immune response in COVID-19 severe infection. (**a**) Network built from differentially expressed datasets related to a hyperinflammatory/immune response obtained by the Gene Set Enrichment Analysis (GSEA) of Low_ACE2 vs. High_ACE2 expressing cell lines. Datasets overexpressed (**b**–**f**) or underexpressed (**g**) in High_ACE2 cell lines. Vertical bars represent where the members of the gene set appear in the list of ranked genes. Genes are ranked on the base of their differential expression in 'Low_ACE2' vs. 'High_ACE2' samples, with genes decreasingly overexpressed in 'Low_ACE2' samples starting from the left of the graph. IL1A (**h**), IL1B (**i**), IFNA21 (**j**) and IFNW1 (**k**) expression in Low_ACE2 vs High_ACE cell lines. FC: expression ratio of High_ACE2 vs. Low_ACE2 cell lines. FC: expression ratio of each transcript in High_ACE2 vs. Low_ACE2 cell lines. Values around the median in (**j**) and (**k**) are compressed toward the bottom because they possess mostly a zero value.

A visualization of the GSEA results using the EnrichmentMap software^24^, a tool that allows to filter out redundancy by grouping together similar gene sets, identified, among the other networks, a 33-node cluster hinting to an immune response in cells overexpressing ACE2 (Fig. 2a). Complex regulatory networks coordinate a controlled immune and inflammatory response in the case of injury or infection^25^. They involve the production of eicosanoids from arachidonic acid and related polyunsaturated fatty acids (i.e., molecules like prostaglandins, leukotrienes and thromboxanes). Several elements of this response are visible in the right part of the cluster in Fig. 2a–d. Recently, an eicosanoid storm has been proposed to be central to tissue damage and multi-organ failure induced by SARS-CoV-2 infection in COVID-19^26^. Accordingly, gene sets whose protein products are targets either of NSAIDs (such as indomethacin, and diclofenac, naproxen, salicylic acid) or other anti-inflammatory compounds (e.g., oltipraz, 2-propenoic acid, 2 phenyl (cinnamic acid), isoprenoids/terpenoids, glyburide and muraglitazar), are present in our network and/or GSEA analysis (Fig. 2a,e,f, Supplementary Fig. 1a–c, Supplementary Table 3). It is interesting to note that only a minority of the edges (circles) of the anti-inflammatory compounds are connected on the network, with a few of them (namely, naproxen and salicylic acid) not even being part of it. This suggests that these compounds have a significant portion of non-overlapping molecular targets and, therefore, their combined therapeutic use should be, in principle, feasible.

According to its role in the inflammatory response^27^, several targets of retinoic acid (RA) metabolism are upregulated, with the top 10 genes shown in Supplementary Fig. 1d. Figure 1d depicts the presence of several inhibitors of RA function, such as DHRS3, a molecule known to attenuate RA signaling^28^, AKR1C3, known to trigger a decrease in the RA biosynthesis flow through its retinaldehyde reductase activity^29^, or members of the CYPs family, that inactivate RA via P450 oxidation^30^. In Fig. 2a, it is also visible the direct association between the ‘GO_RETINOL_METABOLIC_PROCESS’ node with the ‘GO_FLAVONOID_METABOLIC_PROCESS’, suggesting a partially overlapping function. Recently, flavonoids have been proposed as a complementary approach to conventional therapy of COVID-19, either alone^31^ or in combination with vitamin C^32,33^.

### Dexamethasone and the protein folding response

It is known that SARS-CoV-2 infection causes tissue damage, which triggers the endoplasmic reticulum (ER) stress response and subsequent eicosanoid and cytokine storms^26^. The recently approved COVID-19 anti-inflammatory agent dexamethasone, indeed, stimulates resolution of airway inflammation by promoting protein folding and degradation of misfolded proteins from the ER^34,35^. In keeping with the overall results of our model, the protein folding response appears to be already heavily deteriorated in ACE2 overexpressing cells, as shown in Fig. 2g, reinforcing the rationale for the use of therapeutic agents aimed at rescuing its function in this class of patients.

### Interleukin-1 and interferon type 1 responses

Another major theme in the issue of COVID-19 immune response ‘flaring out of control’ is based on the hyperinflammation caused by an increase in proinflammatory cytokines, such as IL-1 and IL-6^36^. Significantly, inhibition of IL-1 function by using the IL1-receptor antagonist anakinra, reduces both the need for invasive mechanical ventilation and the mortality in patients with severe forms of COVID-19^37,38^. The expression of both forms of IL-1, IL1A and IL1B were analyzed in our model, with the result that they were found to be both overexpressed in ACE2 overexpressing cells (Fig. 2h,i), in keeping with the clinical evidence. Instead, blocking the action of circulating IL-6 by tocilizumab has given so far controversial results^39,40^. Accordingly, no evidence of overexpression of IL-6 was found in the model (Supplementary Fig. 2a). An additional emerging issue in the pathogenesis of COVID-19 disease stems from observations that have defined a protective role for type I interferon (IFN) pathways against life-threatening coronavirus disease^41,42^. In humans, the type I IFN system is a family of cytokines consisting of 13 IFN alpha (IFNA) subtype genes, one IFN beta gene (IFNB), one IFN-Epsilon gene (INFE), one IFN-Kappa gene (IFNK) and one IFN-Omega gene (IFNW1). Recently, neutralizing autoantibodies against type I IFNs, mainly IFNA2 and IFNW1, have been identified in up to 13.7% of patients with life-threatening COVID-19 pneumonia, and were shown to be able to impair the capability to block the viral infection of the corresponding antibody^43^. On this premise, we analyzed the involvement of type I IFNs in our model. Results were largely reminiscent of the aforementioned clinical study, with significantly diminished levels of both IFNA2 and IFNW1 in ACE2 overexpressing cells (Fig. 2j,k) and no significant depletion of all other cytokines, but IFA21 (Supplementary Fig. 2b–o). While these results nicely parallel those of Bastard and colleagues^43^, they further suggest the pre-existence of cell-intrinsic, host-dependent predisposing factors in patients with severe COVID-19.

### Other pathways correlated to high ACE2 levels

Supplementary Fig. 3a–d depicts the link of ACE2 overexpression to keratinization/cornification. Indeed, also this pathway is related to the inflammation process and it has been shown that cornification is preceded by the activation of a keratinocyte-specific group of pyroptosis-related genes^44^. Pyroptosis is a cell death pathway activated by a high inflammatory state often occurring upon infection with intracellular pathogens, and it has been recently linked to SARS-CoV-2 infection and even proposed as a therapeutic target in patients with COVID-19^45^. Interestingly, pyroptosis is also a mechanism of IL1B production^46^ and might be contributing to its sustained amounts in ACE2 overexpressing cells, observed also at transcriptional level (Fig. 2i). Other significantly overexpressed datasets also hinted at an involvement in SARS-CoV-2 infection; among those, the intestinal absorption (Supplementary Fig. 3e), with absorptive enterocytes known to be targeted by SARS-CoV-2^12^, mucins overexpressing datasets (Supplementary Fig. 3f), possibly related to the involvement of mucins in the phenomenon of silent hypoxia of patients with COVID-19^47^ and linoleic acid metabolism (Supplementary Fig. 3g), linked to the production of proinflammatory arachidonic acid, previously shown to be relevant for the replication of HCoV-229E, another human coronavirus^48^.

### Pathways correlated to low ACE2 levels

**Figure 3:**
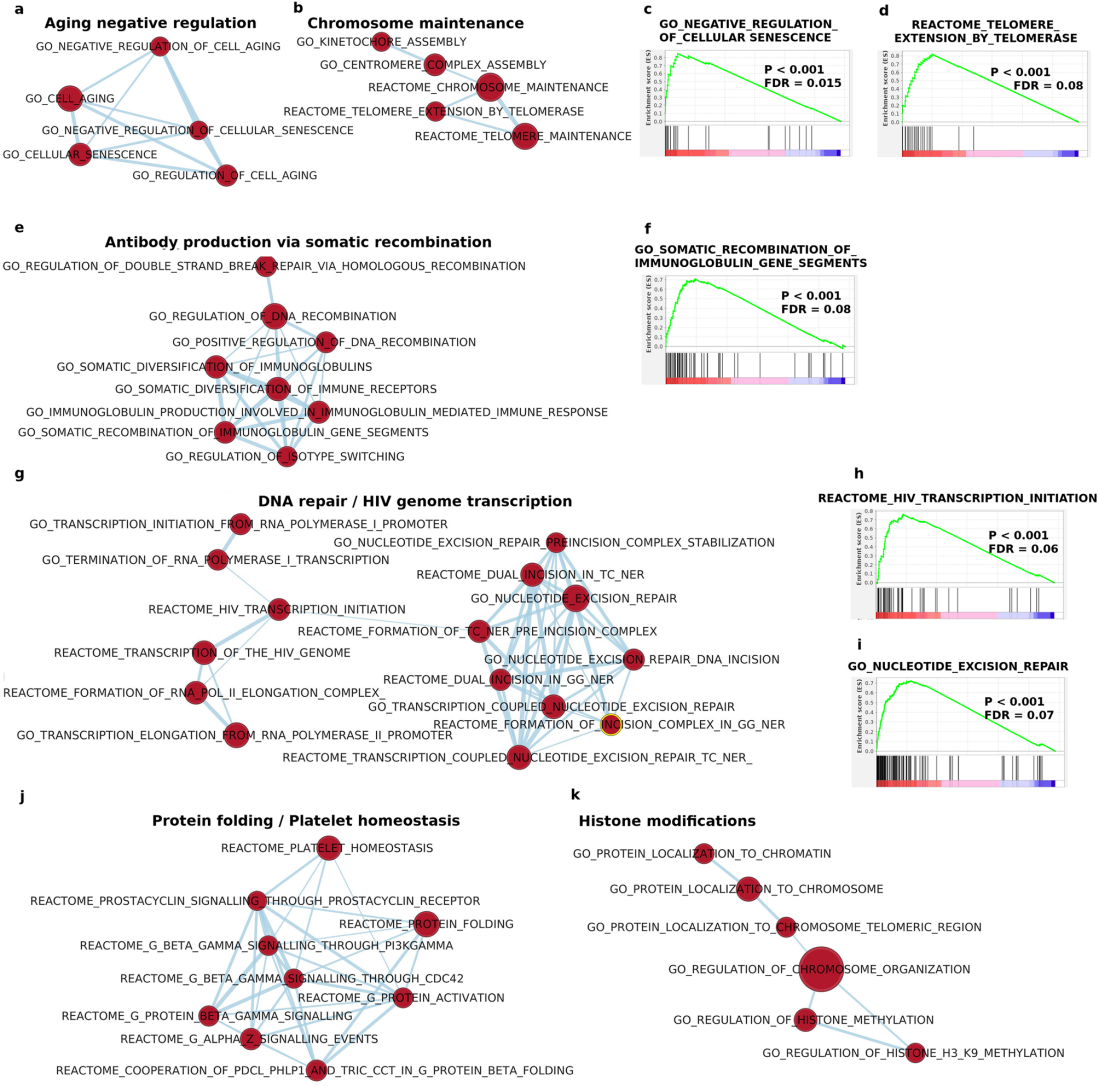
Impairment of several key pathways in ACE2 overexpressing cell lines. GSEA identified several gene sets downregulated in ACE2 overexpressing cell lines. Gene sets could be grouped in pathways/networks related to senescence/chromosome maintenance (**a**–**d**), antibody production (**e**,**f**), DNA repair/viral transcription (**g**–**i**), protein folding/platelet homeostasis (**j**), histone modifications (**k**).

A number of pathways linked to important cellular functions were found to be, instead, correlated to low ACE2 levels, meaning that the underlying function was likely decreased or even missing in ACE2 overexpressing cells. Among these deteriorated functions, we have: aging control and chromosome maintenance (Fig. 3a–d), antibody production (Fig. 3e,f), DNA repair/HIV genome transcription (Fig. 3g–i), protein folding/platelet homeostasis (Fig. 3j), histone modifications (Fig. 3k), apoptosis (Supplementary Fig. 4a–c) and microtubule depolymerization (Supplementary Fig. 4d,e). Taken together, these data point to the presence of several further 'Achille heels' in ACE2 overexpressing cells, reinforcing the idea that a clinically compromised situation might be existing long before viral infection in severe COVID-19.

### Collapsing androgen activity in male highly expressing ACE2 cells

**Figure 4:**
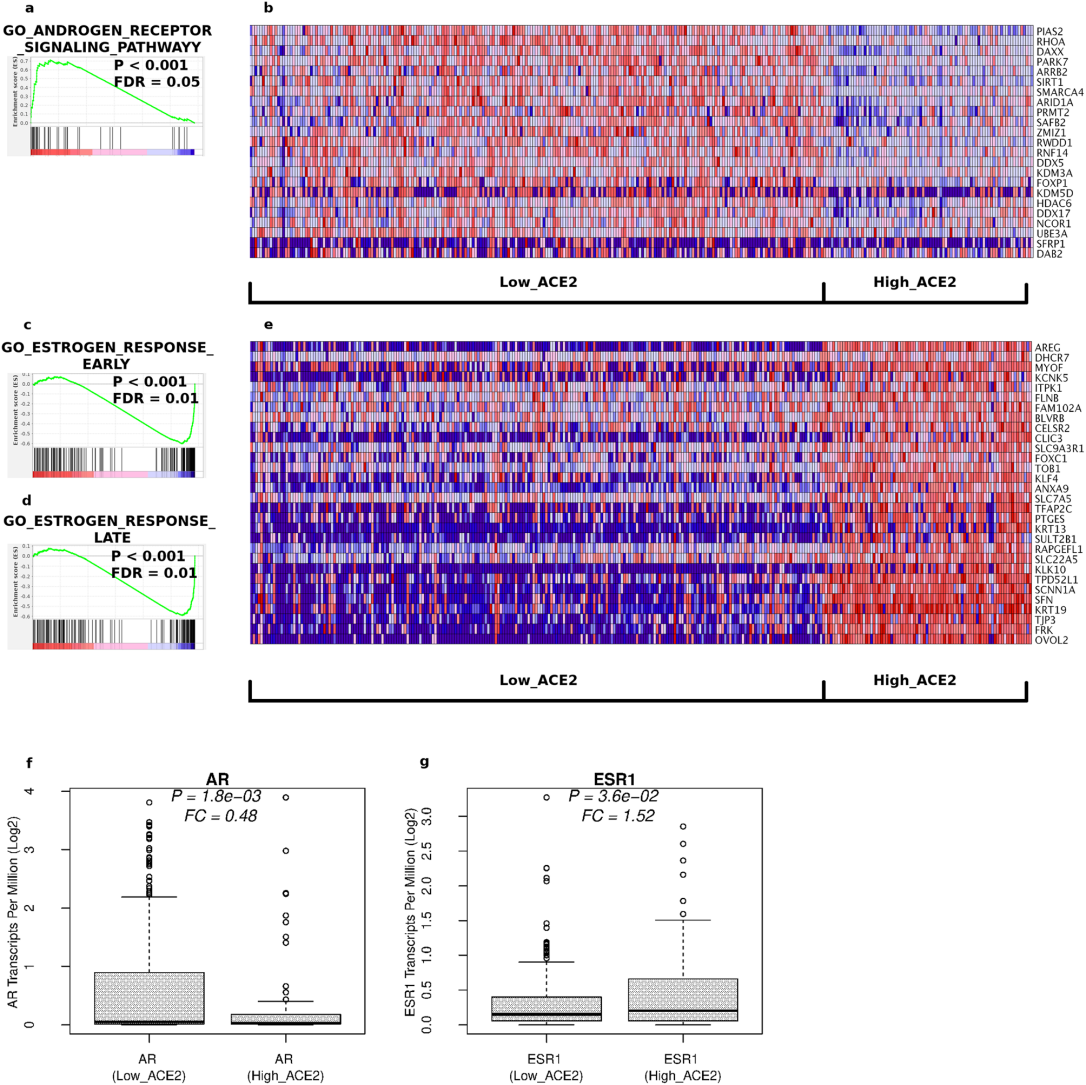
Hypogonadism and estrogen response is coupled to ACE2 overexpression in male cell lines. GSEA in male cell lines identifies a downregulation of the androgen receptor signaling pathway (**a**,**b**), coupled to un upregulation of the estrogen response (**c**–**e**) and to the transcriptional downregulation of the androgen receptor (**f**) and to the transcriptional activation of the estrogen receptor (**g**).

Another emerging issue in severe Coronavirus infections is the phenomenon of a collapsing androgen activity^49^. Indeed, a few studies have clearly demonstrated a reduction of circulating testosterone levels after SARS-CoV-2 infection, with lower testosterone levels predicting the worst clinical outcomes^50,51^. In order to determine if our model could recapitulate also this circumstance, a GSEA search was performed using only the male component of our cell line dataset (n = 310, 230 Low_ACE2 vs. 80 High_ACE2). As a result, the androgen receptor signaling pathway was significantly decreased in ACE2 overexpressing cell lines (Fig. 4a). The heatmap of the significantly decreased genes is shown in Fig. 4b. As a few examples, SIRT1, required for fertility in mice, takes part in acrosome biogenesis, in the differentiation of spermatogenic stem cells and in histone-to-protamine transition during spermatogenesis^52^ or RHOA, a known mediator of clinically relevant androgen action in prostate cancer^53^. GSEA analysis also determined that the same ACE2 overexpressing cell lines had a concomitant, strong estrogen response (Fig. 4c,d), in keeping with the fact that reduced testosterone levels can be caused by its conversion to 17β-estradiol by the enzyme aromatase^54^. The up-regulation of several key genes of this pathway in ACE2 overexpressing cells was also visible (Fig. 4e). Strikingly, even a decrease of the transcription activity of the androgen receptor, coupled to an increase of the transcription of the estrogen receptor, was found in these same cells (Fig. 4f,g). Taken together, these data suggest that the silencing of the androgen response and the activation of estrogen response in patients with COVID-19 might occur at multiple levels, with low testosterone levels likely to be pre-existing in patients with the most severe clinical outcomes. Also in this case, it is conceivable that reinstating patient adequate hormonal levels might be an effective, preventive therapeutic strategy in limiting SARS-CoV-2 infection detrimental effects.

### Model recapitulation of the sex disparity in clinical outcomes

**Figure 5:**
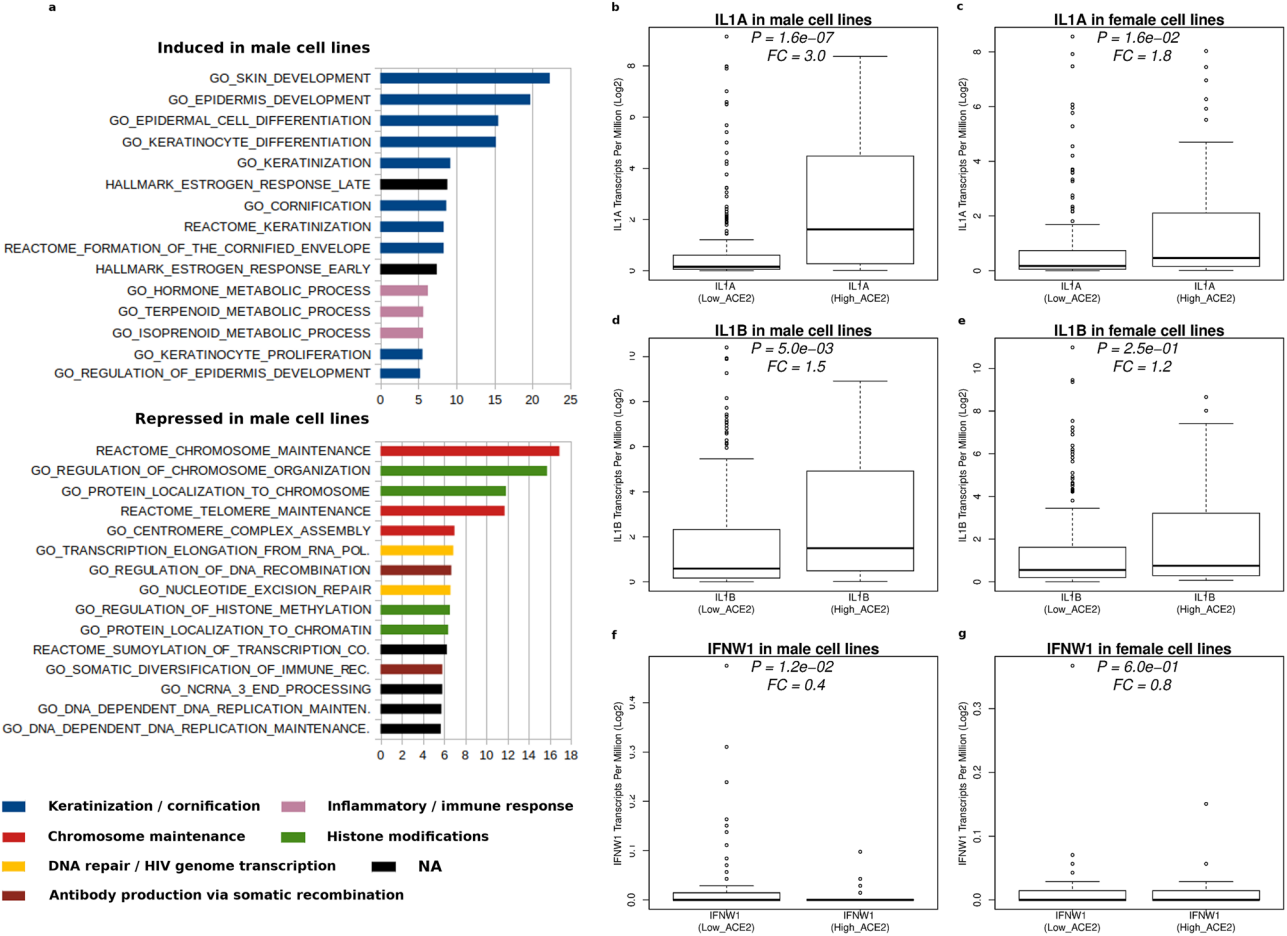
Gender-preferential pathway expression links ACE2 overexpression to worse clinical outcome in males. Measurement of the gender specific activation of the pathways associated to ACE2 overexpression identified 131 gene sets with significant gender preferential activity, with the top 30 lists shown in (**a**). Disease-linked genes IL1A (**b**,**c**), IL1B (**d**,**e**) and IFNW1 (**f**,**g**) also show preferential activation in males.

Besides the issue of low androgen levels, a remarkable general feature of the SARS-CoV-2 infection-associated pandemic is that clinical outcomes are still more severe in men than in women^8,55^. In this context, a variety of factors have been implied, including differences in biology^56^, but also in compliance with public policy rules^57^. We wondered if any of the several infaust, pre-existing conditions we found associated with ACE2 overexpressing cells displayed some sort of sex-related preference. In order to perform this analysis, the degree of activation of the 178 pathways differentially expressed in ACE2 overexpressing cell lines (Supplementary Table 3) was examined separately in male and female cell lines. Then, for each geneset, the activation fold change of their transcripts was calculated and the resulting average activation was compared among sexes (Supplementary Table 4). Figure 5a depicts the top 30 activated pathways. Accordingly, it is clear that, in ACE2 overexpressing cell lines, the impairment to activate pathways linked to tasks like antibody production, chromosome maintenance, DNA repair, etc. is more pronounced in male cell lines. Viceversa, the activation of gene sets linked to the immune response, keratinization/cornification, estrogen response, is less evident in female cell lines. A more detailed example of differential sex-related pathway activation is shown in Supplementary Fig. 5a (chromosome maintenance) and in Supplementary Fig. 5b (regulation of DNA recombination). The disadvantage associated with male sex is also consistently evident for the less significantly differentially expressed gene sets, like for example those belonging to the ‘protein folding/platelet homeostasis’ and to the 'Apoptosis / DNA fragmentation' network, which are less hurted in female cell lines (Supplementary Table 4).

### Interleukin-1 and interferon type 1 responses

A potential sex-related difference in terms of inflammatory cytokine IL-1 increase or IFNA2 and IFNW1 expression decrease was also investigated (Fig. 2h–k). Though with different significance, both IL1A and IL1B were found to be preferentially activated in males (Fig. 5b–e). According to its protective role in the disease^43^, also IFNW1 expression declined more sharply in males (Fig. 5f,g), whereas no difference was found for IFNA2 expression between sexes (data not shown), although this latter analysis was hindered by the low number of available samples with detectable IFNA2 levels (Fig. 2l).

As a whole, our model clearly depicted a different vulnerability among sexes, with males getting the worst of it in almost all differentially expressed pathways.

## Discussion

This study indicates that high ACE2 expression not only promotes infection of cells by SARS-CoV-2, but is also associated with cell-intrinsic characteristics predisposing to the development of a more severe disease phenotype upon infection.

### Pluses and minuses of our model

No ab-initio source-tissue selection of the cell lines was made, with the specific purpose to gain in statistical power and to model the different scenarios related to the several tissues viral entry points^58–60^ and multi-organ effects^61^. Previous work had already identified differential pathways related to ACE2 expression in normal and tumor tissues from lung and other organs in GTEx^62^ and in The Cancer Genome Atlas (TCGA)^10,16^. While advantages of using in-vivo vs. ex-vivo material are quite obvious, the present approach also has some strengths. For example, the stromal component of a tumor tissue can have a confounding effect on the measurement of gene expression, leading to mixed results. Conversely, in our study, besides using clonal cell lines, a robust filtering was made in order to consistently separate low- vs. high-expressing samples, even at the cost of removing more than half of the cell lines from the analysis (Fig. 1a). Despite these differences, there were some overlaps between the findings of the two analyses, since both analyses found that highly expressing ACE2 cells or tissues were susceptible to an inflammatory response, with in-vivo studies also showing that the increased inflammatory signaling was related to higher ACE2 expression in smokers^16^. On the other hand, our model also showed that an eicosanoid storm was likely involved in the ongoing inflammatory response.

### Pathway impairment detection in ACE2 overexpressing cells

Our model also depicted several other landmarks of an advanced disease. This included a decreased protein folding response, supporting the use of dexamethasone (a drug known to ameliorate the protein folding response) in critically ill patients. Likewise, we found that the inflammatory response in ACE2 overexpressing cells was likely to be mitigated by NSAIDs and other anti-inflammatory drugs and compounds, since a significant number of their targets were found to be upregulated. Interestingly, one of the identified NSAIDs, indomethacin, had been already shown to mitigate the effects of SARS-CoV-2 infection both in-vitro and in-vivo^63^. This suggests that the repurposing of NSAIDs for COVID-19 treatment could be an effective therapeutic strategy, especially now that the initial concerns about their use in the specific setting of COVID-19 patients have been retracted^64^. It should be also noted that two anti-inflammatory drugs we found, glyburide and muraglitazar, have been approved to be used in diabetes, a comorbidity known to represent a risk-factor for severe complications in patients with COVID-19^65^. Finally, the involvement of ACE2 overexpression both in establishing a baseline ground for a pathological inflammatory response and in facilitating SARS-Cov-2 infection is becoming increasingly clear from recent studies about the role of smoking in SARS-CoV-2 infection. Indeed, after some controversial results^66^, it is accumulating evidence that the patient's smoking status might have a detrimental effect on the severity of the disease^67^. In these studies, it has been shown that ACE2 is expressed in a population of secretory cells in the respiratory tract. Chronic smoke exposure causes the growth of this cell population, paralleled by an increase in ACE2 expression, whereas quitting smoking reduces the abundance of these respiratory cells and downregulates ACE2 levels^16^. These data are in keeping with the fact that smokers are particularly susceptible to severe SARS-CoV-2 infections. Moreover, since ACE2 expression is upregulated also by viral infection, it is conceivable that SARS‐CoV‐2 invasion could initiate a positive feedback loop, leading to an increased viral dissemination^16^. Interestingly, the overexpression of eicosanoids we found associated in this study to cells with high ACE2 levels regardless of their SARS-CoV-2 infection, were found to be diminished in recovered COVID-19 patients^68^, further underlining the virus capability to exacerbate pre-existing morbidity conditions.

Other compromised pathways in ACE2 overexpressing cells pointed to an impairment in both senescence control and chromosome maintenance, in agreement both with epidemic data showing correlation of ACE2 expression with age^7^ and with the demonstrated greater vulnerability to SARS-CoV-2 in elderly people^8–10^. Overexpressing ACE2 cell lines displayed also several other weaknesses, like: (a) A reduced capability to produce immunoglobulins via somatic recombination, reinforcing the rationale for potential therapeutic approaches using monoclonal antibodies or plasma of recovered patients containing neutralizing antibodies, as an effective treatment option to decrease the viral load and to reduce mortality^69,70^; (b) An attenuated power in repairing damaged DNA, a pathway already known to be hijacked by the HIV virus for initiating transcription without occurring into the host innate immune sensing^71^, suggesting that also SARS-CoV-2 might exploit similar routes to perform immune-escape; (c) A decreased activity of pathways linked to life-threatening conditions like the prostacyclin signaling, leading to platelet homeostasis, needed to overcome the hypercoagulable state observed in patients with COVID-19^72^; (d) A reduced capability to perform histone modifications, a process deeply linked to aging^73^ and to COVID-19 severity^74^; (e) A decreased apoptotic capability. Apoptosis is a programmed process of cell death in which damaged cells are removed without triggering inflammation^75,76^. It is often working less efficiently during aging, resulting in the accumulation of malfunctioning cells within an organism, ultimately contributing to its senescence^77,78^; (f) A decreased capability to depolymerize microtubules. Interestingly, it has been shown that the intracellular transport of viral particles is indeed mediated by microtubules and associated proteins^79^; as a consequence, when using agents like colchicine to inhibit microtubule polymerization, it is possible to observe a significant decrease in the replication of viruses like dengue and Zika^80^. On this premise, clinical trials employing colchicine in the treatment of COVID-19 patients have also started^81^.

### Hypogonadism and recapitulation of the sex disparity in clinical outcomes

Another important validation of our model comes from its ability to predict collapsing androgen levels related to elevated ACE2 expression in the male component of our dataset. Diminishing androgen activity is an important, emerging issue in male patients with severe COVID-19, whose clinical outcome seems to be significantly correlated to their testosterone levels^50,51^. Recently, our group also uncovered an independent association between COVID-19 infection status and testosterone levels at hospital admission in a relatively large case–control study, with lower testosterone levels correlating with the most severe clinical outcome^82^. Several reasons could account for this association. For instance, low testosterone levels might simply be a marker of disease severity. Alternatively, an acquired condition of low testosterone—which characterizes up to 20% of middle‐aged/elderly men^83^—might promote an overall greater incidence, higher severity, and greater chance of lethal events in men compared to women. It should also be considered that the testosterone-producing Leydig cells possess high ACE2 amounts^84^. This, in principle, could favour their SARS‐CoV‐2 invasion, ultimately leading to a functional dysregulation in their testosterone production. In our model, it was also clearly visible a spike in the activation of the estrogen response, a phenomenon usually associated with decreasing testosterone levels^54^. Likewise, the transcriptional activity of both androgen and estrogens receptors was also found to be modified; indeed, while it was decreased for the androgen part, it emerged to be increased for the estrogen counterpart, thus suggesting that the shutting down of the whole androgen pathway in at-risk patients, could occur not only at the post-transcriptional level, but also be part a much wider cellular orchestration. In keeping with the silencing of the androgen function in males, we found that several other pathways affected preferentially men rather than women, recapitulating also this relevant aspect of the disease. It should be also noticed that ACE2 is a key regulator of the renin-angiotensin system implied in the regulation of cardiovascular and renal function^85^, where also significant disparities in the clinical outcome are observed^86^.

### Future perspectives

We have done a rather limited use of gene sets in our analysis, since it was restricted to the following databases: Reactome, Keggs, Biological Processes of Gene Ontology and Hallmarks of the MSigDB. It is conceivable that a more extensive analysis might identify other pathways related to high ACE2 levels and, possibly, to the severity of COVID-19 status. Moreover, a rather stringent cut-off was used for GSEA analysis (p < 0.001). It is conceivable that slightly more relaxed search parameters would have yielded, along with a mild increase in the search background, a broader spectrum of differentially expressed pathways in our analysis. Similarly, since we used a slightly old drug signature database, DSigDB^23^, it is likely that a more up-to-date and/or large-scale signature profile analysis might uncover new compounds repurposable for disease treatment. Another pursuable avenue would consist in replicating the present ‘guilt-by-association’ approach using, instead of ACE2, the recently discovered SARS-CoV-2 entry point neuropilin-1 (NRP1)^58,59^, aiming at identifying also in that case novel patho-physiological conditions, pathways and drugs linked to that kind of SARS-CoV-2 entry modality.

### The SARS-CoV-2 infection as a tipping point

Finally, besides validating a new, surprising disease model, this study underlines the concept that a variety of pre-existing pathological conditions can prime the severity of SARS-CoV-2 infections, which eventually become a tipping point at which they become clinically evident. Therefore, while we wait for an effective vaccine, in order to decrease casualties, it would be of paramount importance to defend the population at risk. This should be done not only by minimizing their exposure to the virus, but also by actively improving their general well-being by taking measures that will reduce, whenever possible, their risk factors, including those outlined in this study.

## Methods

Log2 transformed TPM (Transcripts Per Million) RNA-seq data of all transcripts of 1305 human cancer cell lines from the Cancer Cell Line Encyclopedia were downloaded from the Depmap portal (https://depmap.org/portal/). Cell lines displaying an ACE2 content equal to 0 TPM (Transcripts Per Kilobase Million) (n = 427) (‘Low_ACE2’) or equal or higher than 1 TPM (n = 169) (High_ACE2), were selected for differential gene expression analysis using the limma package (http://www.bioconductor.org/packages/release/bioc/html/limma.html), employing a fold-change cutoff of 1.5 and a multiple hypothesis, Bonferroni-corrected P-value threshold of 1e−05. This rather robust separation of the samples (P = 6.3E−157) was aimed at defining two clearly differentiated populations for the analysis. Therefore, while the Low_ACE2 group was chosen as the one with a zero ACE2 TPM content, the lower bound of the High_ACE2 group, greater than 1 TPM, was chosen as the minimal value that, while keeping separated as much as possible the samples, was yielding a sample size with enough statistical power to detect, if present, a number of differentially expressed genes comparable to the ones found to be correlated to ACE2 expression in a similar study on human tissues^87^. This high degree of sample separation has been found to be especially important when analyzing expression data for relatively low abundance transcripts, such as ACE2, coming from microarray^88^ or RNA-seq experiments^89^.

Biological processes Gene Ontology analysis reported in Supplementary Fig. 1 and Supplementary Table 2 was performed by inputting to TermFinder program^90^ the top 50 regulated transcripts in ‘High_ACE2’ samples from the CCLE dataset.

Gene Set Enrichment Analysis (GSEA) was performed first using the whole Low_ACE2 vs. the High_ACE2 dataset vs. the following gene sets: Reactome and the Kegg databases from the C2 Molecular Signature Database (MSigDB), the Gene Ontology Biological Processes database from the C5 MSigDB and the Drug Signature Database (DsigDB) version 1.0. Stringency cutoff for GSEA searches were: P-value < 0.001, FDR <  = 0.1. In order to capture also gender-specific pathways, the original 596 cell lines dataset was split in two datasets, according to the gender source (https://ndownloader.figshare.com/files/25494443): females (n = 226, 154 Low_ACE2 vs. 72 High_ACE2) and males (n = 310, 230 Low_ACE2 vs. 80 High_ACE2). A fraction of cell lines (n = 60) was found to be of unknown source and was removed from the search. Then, independent GSEA searches were performed using either the male or the female or the male plus female input datasets. Conditions were the same as before, except that the FDR was set to <  = 0.05. Analyses were done using the GenePattern suite of programs (http://www.genepattern.org). Results were downloaded and imported into the Enrichment Map^24^ plugin for Cytoscape^91^ for network analysis using default values. The DsigDB database version 1.0 was obtained from http://dsigdb.tanlab.org/DSigDBv1.0/.

Differential activation of genesets among sexes was determined by calculating, for each gene of the male and female datasets, the log twofold change between Low_ACE2 and High_ACE2 samples. From each gene pair of these datasets showing significant differential expression (FDR <  = 0.05) in the male and/or in the female dataset, a sub dataset was built in order to perform a paired t test on the logarithms. The activation fold change of each sub dataset was calculated as the antilogarithm of the difference between logarithms.

### Outline of tools used in this study

DepMap portal (https://depmap.org/portal/).

GenePattern (https://www.genepattern.org/).

Gene Set Enrichment Analysis (https://www.gsea-msigdb.org/gsea/index.jsp).

Molecular Signature Database (https://www.gsea-msigdb.org/gsea/msigdb/index.jsp).

Drug Signature Database (http://dsigdb.tanlab.org/DSigDBv1.0/).

R software package (http://www.r-project.org).

## Supplementary Information

Supplementary Information 1.

Supplementary Table S1.

Supplementary Table S2.

Supplementary Table S3.

Supplementary Table S4.

## Data Availability

Expression data for the 1305 human cell lines from the CCLE project used in this study are available at this link: https://ndownloader.figshare.com/files/24613349. The open source R software package is available at http://www.r-project.org. Scripts and data used for generating figures are available as Supplementary Data. All the other data are available from the corresponding author upon reasonable request.
