# Supplementary material for: A virus-free cellular model recapitulates several features of severe COVID-19
Source: Sci Rep. 2021 Sep 1;11:17473. doi: 10.1038/s41598-021-96875-7 (PMC8410838; doi:10.1038/s41598-021-96875-7)
Supplement: Supplementary file 1 — Supplementary Information 1. [file 41598_2021_96875_MOESM1_ESM.pdf]

Supplementary Information

A virus-free cellular model recapitulates several features of severe COVID-19

Giovanni Lavorgna*[1], Giulio Cavalli[2,3], Lorenzo Dagna[2,3], Silvia Gregori[4], Alessandro Larcher[1], Giovanni Landoni[2,5], Fabio Ciceri[2,6], Francesco Montorsi[1,2], Andrea Salonia[1,2]

[1]Division of Experimental Oncology/Unit of Urology; URI; IRCCS Ospedale San Raffaele, Milan, Italy

[2]University Vita-Salute San Raffaele, Milan, Italy

[3]Unit of Immunology, Rheumatology, Allergy and Rare Diseases (UnIRAR), IRCCS San Raffaele Scientific Institute, Milan, Italy

[4]San Raffaele Telethon Institute for Gene Therapy (SR-TIGET), IRCCS Ospedale San Raffaele, Milan, Italy

[5]Anesthesia and Intensive Care Department, IRCCS Ospedale San Raffaele, Milan, Italy

[6]Hematology and Bone Marrow Transplant Unit, IRCCS Ospedale San Raffaele, Milan, Italy

***Corresponding author:** lavorgna.giovanni@hsr.it

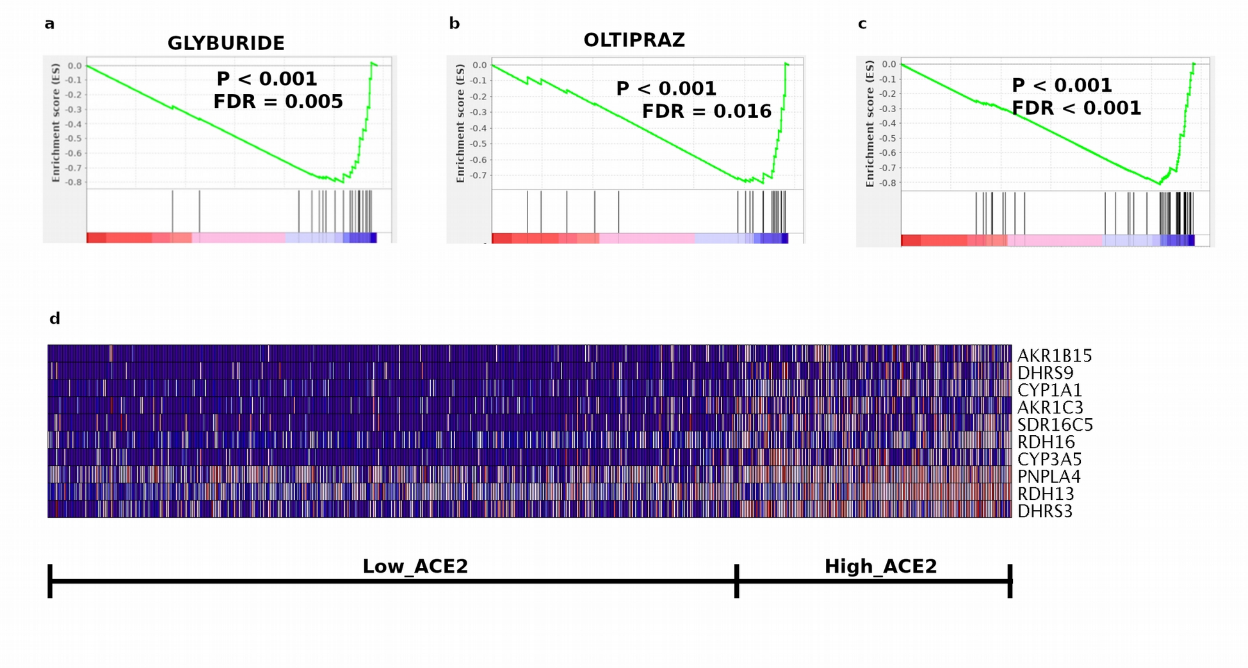

Supplementary Fig. 1

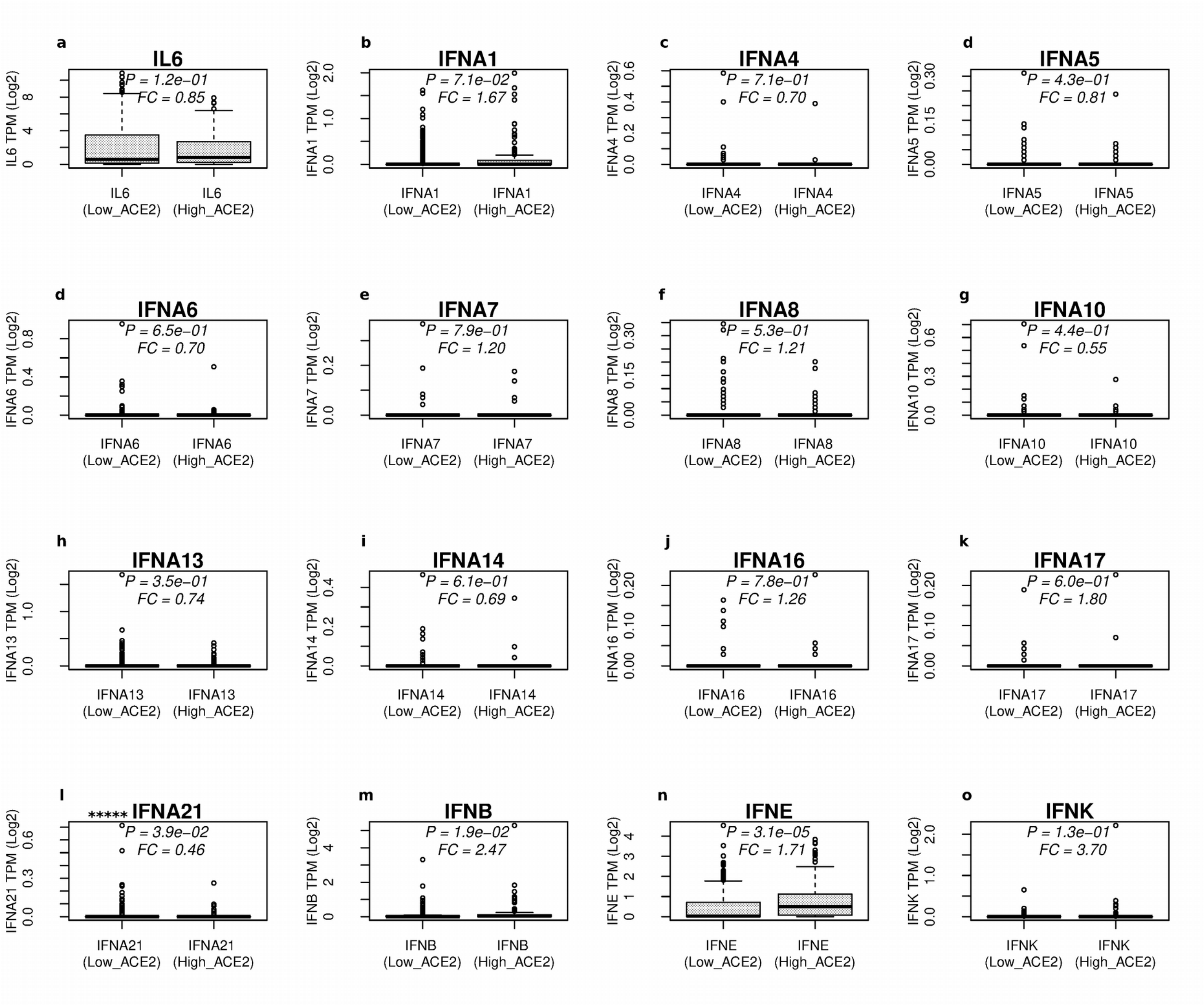

Supplementary Fig. 2

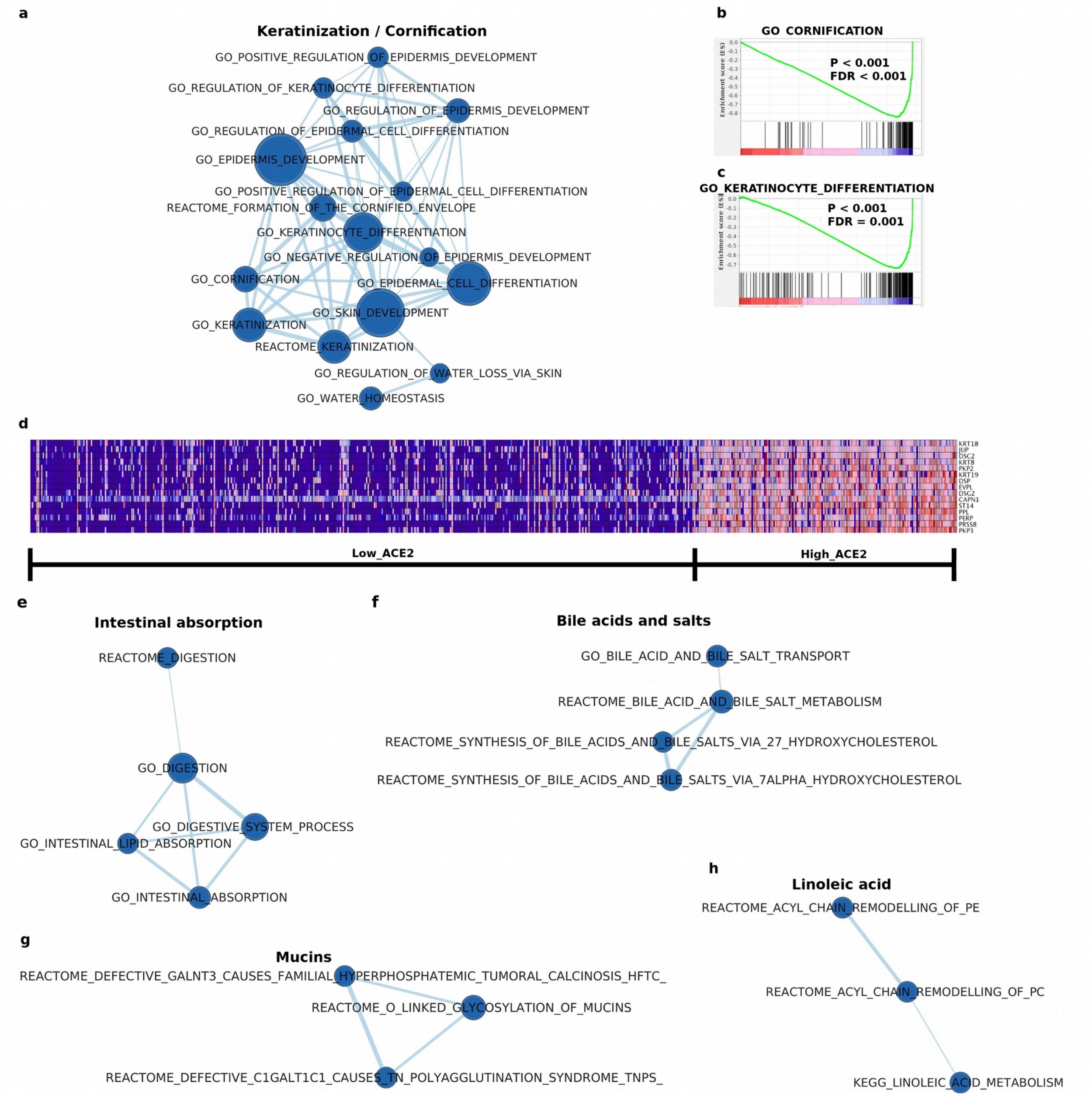

Supplementary Fig. 3

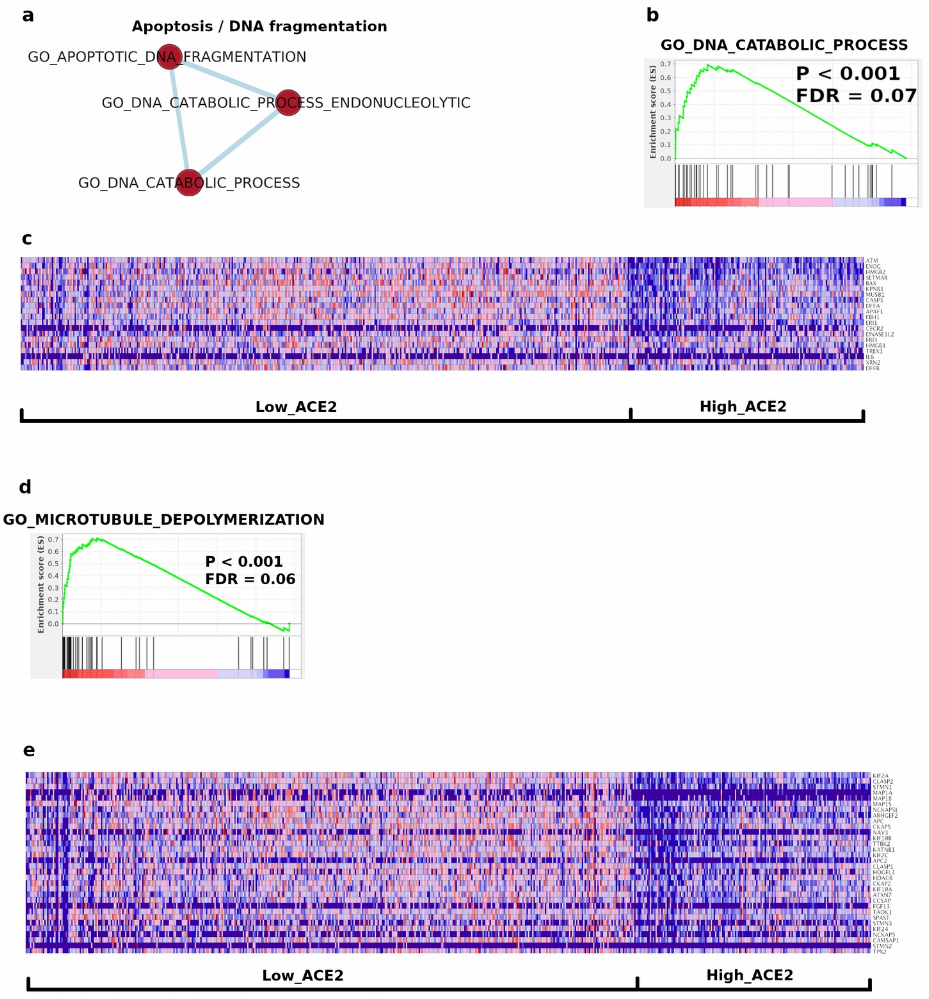

Supplementary Fig. 4

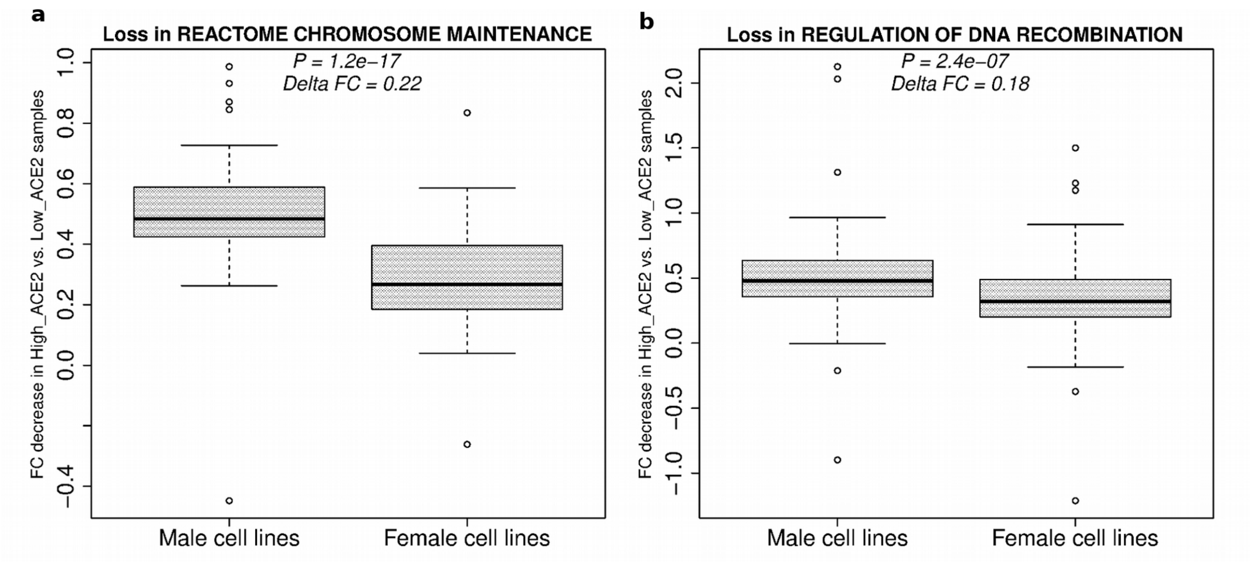

Supplementary Fig. 5

Legends to Supplementary Figures

Supplementary Figure 1. Extended features of the molecular pathways linked to ACE2 overexpression in cell lines. (**a-c**) Datasets overexpressed in High_ACE2 cell lines. (**d**) Heat map of the top-10 differentially expressed transcripts in the dataset 'GO_RETINOL_METABOLIC_PROCESS'.

Supplementary Figure 2. Expression of IL6 (**a**) and type I interferon system genes in Low_ACE2 and High_ACE2 cell lines (**b-o**). IFNA21 (**j**) gene plot, showing a significant downregulation in HigH_ACE2 samples, is asterisked. FC: expression ratio of each transcript in High_ACE2 vs. Low_ACE2 cell lines.

Supplementary Figure 3. Additional features of the molecular networks linked to ACE2 overexpression. (**a**) Network of keratinization/cornification datasets overexpressed in High_ACE2 cell lines. GSEA of cornification (**b**) and keratinization (**c**) datasets.(**d**) Heat map of the top-15 differentially expressed transcripts in the dataset 'GO_CORNIFICATION''. (e-h)

Supplementary Figure 4. ACE2 overexpression is linked to both decreased apoptotic capability (**a-c**) and to decreased microtubule depolymerization (**d-e**). Additional networks linked to ACE2 overexpression.

Supplementary Figure 5. Graph representation of differential gender expression of GO_CHROMOSOME_MAINTENANCE (**a**) and GO_REGULATION_OF_DNA_RECOMBINATION (**b**) genesets.

Legends to Supplementary Tables

Supplementary Table 1. Differentially expressed transcripts in 'High ACE2' samples from the CCLE dataset

Supplementary Table 2. TermFinder analysis of the top 50 regulated transcripts in 'High-ACE2' samples from the CCLE dataset.

Supplementary Table 3. Gene sets significantly enriched or depleted in 'High ACE2' samples from cell lines of the CCLE project.

Supplementary Table 4. Gene sets showing preferential gender expression in ACE2 overexpressing cell lines.
